# Analytical Methods Used in Determining Antioxidant Activity: A Review

**DOI:** 10.3390/ijms22073380

**Published:** 2021-03-25

**Authors:** Irina Georgiana Munteanu, Constantin Apetrei

**Affiliations:** Department of Chemistry, Physics and Environment, Faculty of Sciences and Environment, “Dunărea de Jos” University of Galaţi, 47 Domneasca Street, 800008 Galaţi, Romania; georgiana.munteanu@ugal.ro

**Keywords:** antioxidant activity, superoxyde dismutase (SOD), reactive oxygen species (ROS)

## Abstract

The study of antioxidants and their implications in various fields, from food engineering to medicine and pharmacy, is of major interest to the scientific community. The present paper is a critical presentation of the most important tests used to determine the antioxidant activity, detection mechanism, applicability, advantages and disadvantages of these methods. Out of the tests based on the transfer of a hydrogen atom, the following were presented: the Oxygen Radical Absorption Capacity (ORAC) test, the Hydroxyl Radical Antioxidant Capacity (HORAC) test, the Total Peroxyl Radical Trapping Antioxidant Parameter (TRAP) test, and the Total Oxyradical Scavenging Capacity (TOSC) test. The tests based on the transfer of one electron include the Cupric Reducing Antioxidant Power (CUPRAC) test, the Ferric Reducing Antioxidant Power (FRAP) test, the Folin–Ciocalteu test. Mixed tests, including the transfer of both a hydrogen atom and an electron, include the 2,2′-Azinobis-(3-ethylbenzothiazoline-6-sulfonic acid (ABTS) test, and the [2,2-di(4-tert-octylphenyl)-1-picrylhydrazyl] (DPPH) test. All these assays are based on chemical reactions and assessing the kinetics or reaching the equilibrium state relies on spectrophotometry, presupposing the occurrence of characteristic colours or the discolouration of the solutions to be analysed, which are processes monitored by specific wavelength adsorption. These assays were successfully applied in antioxidant analysis or the determination of the antioxidant capacity of complex samples. As a complementary method in such studies, one may use methods based on electrochemical (bio)sensors, requiring stages of calibration and validation. The use of chemical methods together with electrochemical methods may result in clarification of the operating mechanisms and kinetics of the processes involving several antioxidants.

## 1. Introduction

The occurrence of degenerative processes is correlated in molecular biology with the existence of a surplus of free radicals, promoting oxidative processes that are harmful to the body. Plants’ high content of compounds with antioxidant properties able to capture free radicals (carotenoidic, phenolic, flavonic, anthocyanic derivatives, unsaturated fatty acids, vitamins, enzymes and cofactors) has stimulated interest in using them in prophylactic and curative phytotherapy.

The role of the antioxidants is to neutralise the free radicals in biological cells, the free radicals having a negative impact on living organisms. A special role in neutralising the effects of the oxidative stress related to the presence of free radicals is played by the enzyme called superoxyde dismutase (SOD). It is a metaloenzyme with subunitary structural organisation, being the main regulator of the oxidation processes in biological cells. This enzyme catalyses the recombination reaction of the oxygen radicals. Applying antioxidant therapy by using SOD is efficient in treating various pathological states of the human body, as well as in preventing their occurrence (it prevents the formation of hydrogen peroxyde and triplet oxygen).

Oxidative stress is a relatively new concept, widely used in medical sciences in the past three decades. It takes an active part in the physiology of very common diseases, like diabetes, high blood pressure, preeclampsia, atherosclerosis, acute renal failure, Alzheimer’s and Parkinson’s. The cells, through metabolising oxygen, create reactive species of oxygen (ROS), that are potentially harmful. Under normal circumstances, the rate and amplitude of oxidant formation is balanced by the rate of their removal. However, loss of balance between pro-oxidants and antioxidants results in oxidative stress. High levels of ROS in biological cells have a large impact on their functioning, leading to deficient cell operation, aging, or disease [1].

The presumed ROS and non free-radical species are summarised in Table 1.

Numerous studies show that antioxidants play an essential role in maintaining human health, preventing and treating diseases, due to their ability to reduce oxidative stress. Measuring the antioxidant activity/capacity of foods and biological samples is therefore essential not only in ensuring the quality of functional foods, but more importantly in studying the efficiency of food antioxidants in preventing and treating the diseases related to oxidative stress. 

Antioxidants are compounds which, when present in foods or the human body in very low concentrations, delay, control or prevent oxidative processes leading to food quality deterioration or the occurrence and propagation of degenerative diseases in the organism. A number of methods and activities are involved in the process of inhibiting the oxidation by these antioxidant compounds [2]. 

Molecules with antioxidant properties may be produced endogenously or ingested exogenously by diet or food supplements. The main endogenous antioxidant enzymes are SOD, catalase (CAT), and glutathion peroxydase (GSH-Px). SOD converts the superoxide anion to H_2_O_2_, which is a substrate for CAT and GSH-Px. Catalase metabolises H_2_O_2_ in water and oxygen, and GSH-Px reduces both H_2_O_2_, and organic hydroperoxydes when reacting with glutathion (GSH). 

Exogenous antioxidants, like vitamins E and C, may exist in the organism in the cell membrane, and the intracellular and extracellular liquid. They react with ROS to eliminate or to inhibit them. The hydrophobic lipid interior of the membranes requires a different spectrum of antioxidants. Fat-soluble vitamin E is the most important antioxidant in this environment, protecting against loss of membrane integrity. 

Fat-soluble antioxidants are important in preventing the peroxidation of polyunsaturated fatty acids (PUFA) in biological membranes. Water-soluble antioxidants, like vitamin C, play a key role in neutralising ROS in the hydrophilic phase.

This review provides a general and up-to-date overview of methods available for measuring antioxidant activity and the chemistry principle behind them. In addition, the most important advantages and shortcomings of each method were analysed and highlighted. Understanding the principle mechanisms, advantages and limitations of the measurement assays is important for proper selection of method(s) for valid evaluation of antioxidant potential in practical applications.

## 2. Classification of Antioxidants

According to their operating mechanism, antioxidants may be classified into primary and secondary antioxidants. The antioxidants inhibit the chain reaction of oxidation, acting as hydrogen donors or acceptors of free radicals, generating stabler radicals. The antioxidants in this group mainly have a phenolic structure and include the following: antioxidant minerals, antioxidant vitamins and phytochemicals, among which there are flavonoids, catechins, carotenoids, β-carotene, lycopene, diterpene and their derivatives. These compounds interact by a variety of mechanisms including binding of metal ions, scavenging reactive oxygen species, converting hydroperoxides to non-radical species, absorbing UV radiation or deactivating singlet oxygen. This category includes: butylhydroxyanisol (BHA), butylhydroxytoluene (BHT) and propyl galate (PG) [3].

The efficacy of antioxidant compounds depends on several factors, the most important being structural properties, temperature, the characteristics of the substrate susceptible to oxidisation, concentration, along with the presence of synergistic and pro-oxidant compounds and the physical state of the system. The chemical structure of an antioxidant determines its intrinsic reactivity to free radicals and other ROS and thus influences antioxidant activity. The efficiency of antioxidants also depends on their concentration and localisation in the system, e.g., the interface distribution [4]. Another factor with an important role in their protective action, in the short or long-term, is the kinetics of the reaction. This involves the reaction rate between an antioxidant and a distinct oxidant, the thermodynamics of the reaction and the antioxidant’s ability to react [5]. All these parameters should be taken into account when considering the efficiency and selection of an antioxidant substance suitable for a particular use.

**Figure 1:**
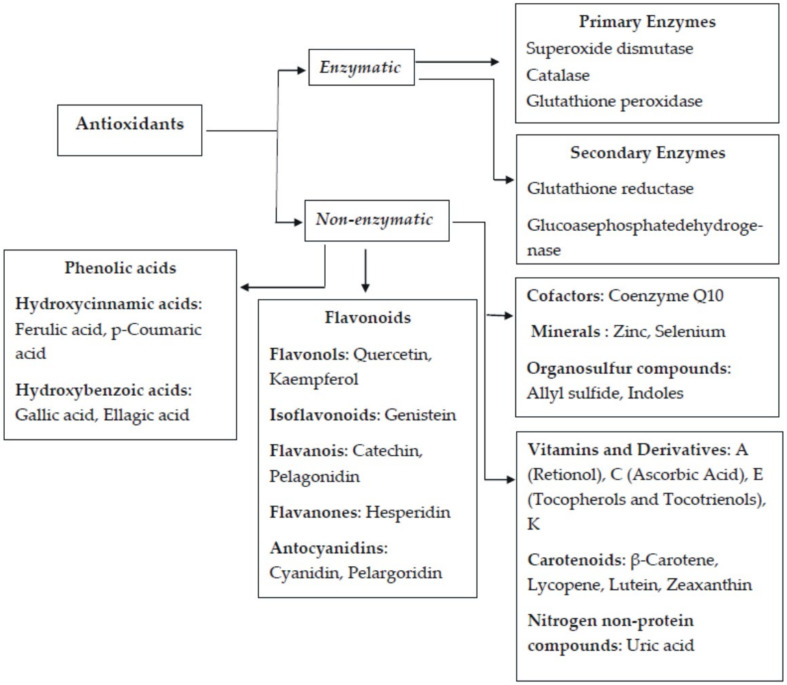
Classification of antioxidants [6].

**Figure 2:**
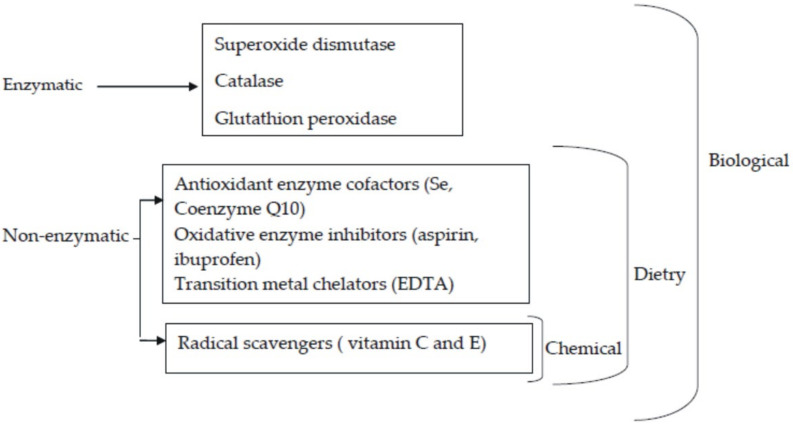
Application scope of antioxidants [7].

Figure 1 illustrates the classification of antioxidants whereas Figure 2 indicates the application scope of antioxidants.

## 3. Measuring the Antioxidant Activity

The methods and instruments used to measure the activity of the antioxidants have made remarkable progress in the past few decades. Early methods measure the efficiency of the antioxidants against the formation of particular species of oxidation products and thus, are based on measuring lipid oxidation. Thus far, various chemical tests coupled with highly sensitive and automated detection technologies have been used to evaluate antioxidant activity by special methods, like for instance scavenging activity against different types of free radicals or ROS, reducing power and metal chelation, among others. Oxidation substrates have also been extended from food model systems to chemical compounds, biological materials, cellular lines and even living tissues [2].

A high number of tests are available for the direct measurement of the transfer of the hydrogen atom or the transfer of electrons from antioxidants to free radicals. The antioxidant activities reported in this method group are generally associated with their capacity to neutralise certain types of radical species, out of which some may be artificial and biologically irrelevant. As a result, these methods have the disadvantage that they do not reflect the situation in an oxidant food or an in vivo case. Nevertheless, the data regarding the hydrogen atom transfer or the data regarding the donating capacity of the electrons obtained by these methods provide important information on their intrinsic antioxidant potential with minimal environment interference. These tests do not require a lipid substrate and normally use a chemical system containing an oxidant (free radicals or other ROS), an oxidising substrate (some tests do not need it) and antioxidants under investigation. 

A standardised method for antioxidant activity of a food component should meet the following ideal requirements [8]:

- The radical source used must be biologically relevant;
- It is desirable for it to be simple;
- The method used must have a defined endpoint and chemical mechanism;
- Both the instruments used and the chemicals must be readily available;
- Reproducibility within the cycle and between days is appropriate;
- It allows analysis for both hydrophilic and lipophilic antioxidants, using different radical sources;
- The method must be applicable for quality control analyses.

It should be emphasised that antioxidant activity must not be tested on the basis of a single method. Several antioxidant procedures should be performed in vitro to determine antioxidant activities for the sample of interest. Taking this into account, it is difficult to compare one method completely with another. Therefore, the methods of analysis must be checked before choosing one for the purpose of research.

The various methods for evaluation of the antioxidant capacity fall into three distinct categories namely, spectrometry, electrochemical assays and chromatography [3] as presented in Table 2.

## 4. Chemical Tests Determining Antioxidant Activity

According to the chemical reactions that may be involved, these tests are divided into two categories: hydrogen atom transfer (HAT) and single electron transfer (SET) reaction-based methods. The end result is the same, regardless of the reaction mechanism involved, although the kinetics and reaction stages are different [8]. The SET and HAT reactions coupled with proton exchange reactions may occur in parallel with the main reactions, and in this case the dominant mechanism in a given system depends on the antioxidant structure and properties, solubility and partition coefficient, as well as the solvent system.

The chemical tests measuring antioxidant capacity are accessible, fast, and typically automated, being used predominantly in screening and initial assessment of new antioxidant compounds or the extracts of final real products/by-products.

### 4.1. Tests Based on the Transfer of the Hydrogen Atom (HAT)

The tests based on the transfer of the hydrogen atom measure the ability of an antioxidant to remove the free radicals by donating a hydrogen atom. The HAT mechanisms of antioxidant action are proven in the following reaction where the hydrogen atom (H) of a phenol (ArOH) is transferred to a peroxyl radical:

ROO^•^ + AH/ArOH ⇄ ROOH + A^•^/ArO^•^

where: the aryloxyl radical (ArO^•^) formed from the reaction of the phenol (ArOH, an antioxidant) with a peroxyl radical (ROO^•^) is stabilised by resonance and AH are the protected biomolecules.

An efficient phenolic antioxidant should react faster than the biomolecules (the protected molecule) with the free radicals in order to give a protective effect against their oxidation [9].

Typical examples of HAT-based tests are the Oxygen Radical Absorption Capacity (ORAC), Total Peroxyl Radical Trapping Antioxidant Parameter (TRAP) and Total Oxyradical Scavenging Capacity (TOSC) assays [10].

#### 4.1.1. The ORAC Test

The ORAC test measures the splitting ability of the radical chain reaction by antioxidants through monitoring the inhibition of the oxidation of the peroxyl radical. Peroxyl radicals are characterised as free radicals that predominate in lipid oxidation in biological systems and also in foodstuffs, under physiological conditions. As a result, ORAC values are appreciated by certain researchers as biologically relevant, a benchmark for antioxidant efficiency.

Commonly used peroxyl radical generators in this assay are represented by azo-compounds, including lipophilic α,α,-azobisizobutyronytril (AIBN), 2,2-azobis(2-amidinopropane) chlorhydrate (ABAP), 2,2′-azobis(2,4-dimethylvaleronytril) (AMVN) and the hydrophilic 2,2′-azobis(2-amidinopropane) dihydrochloride (AAPH) [11].

According to this test, the peroxyl radical emitted by a generator reacts with a fluorescent sample that leads to loss of fluorescence, registered on a fluorimeter. This method uses the area-under-curve technique in the presence and in absence of the antioxidant. As a reference compound is used a standard antioxidant, typically trolox, and the ORAC values of the evaluated antioxidants are described as trolox equivalent. The ORAC test describes the antioxidants’ ability to yield the hydrogen atom and, consequently, it is a HAT-based assay. In order to improve the method, a high-throughput assay has been developed using a multichannel liquid handling system coupled with a microplate fluorescence reader [12].

**Figure 3:**
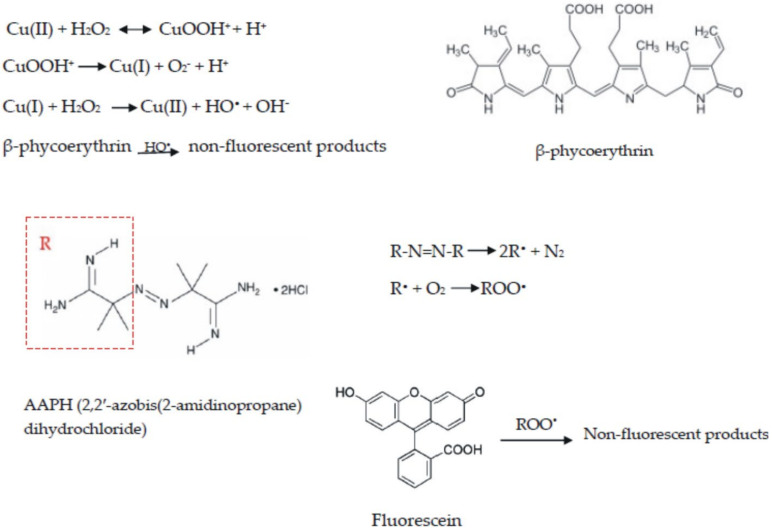
Reaction schemes involved in Oxygen Radical Absorption Capacity (ORAC) assay for the detection of hydroxyl and peroxyl radicals.

The system H_2_O_2_–CuSO_4_ is generally used as a hydroxyl radical generator and β-phycoerythrin used as a redox-sensitive fluorescent indicator protein, whose decay in fluorescence is measured in the presence of free radical scavengers, using Trolox as standard (Figure 3). In the first step, complexation of Cu(II) by H_2_O_2_ leads to the formation of copper(II) hydroperoxide. The latter suffers unimolecular decomposition in the slow step to form Cu(I) and O_2_^−^. In the absence of hydrogen-atom donors, the homolytic cleavage of the Cu–O bond might be the preferred pathway for the decomposition of CuOOH^+^. In the next equation, Cu(I) reacts with H_2_O_2_ to form a hydroxyl free radical.

The azo AAPH compound is the most used peroxyl radical generator in hydrophilic systems, using as fluorescent probe β-phycoerythrin or, more recently, fluorescein. Thus, peroxyl radicals are formed by thermodecomposition of AAPH, giving an alkyl radical that reacts with molecular oxygen to give peroxyl radical. Taking into account the fact that the generation of peroxyl radical is influenced by temperature, it is considered one of the major factors that interfere with the results. Small temperature differences in the external wells of the microplate can decrease the reproducibility of the assay. Thus, it is of major importance to monitor and adjust the temperature during the assay. Nevertheless, the problem was remedied by developing automated fluorescent microplate readers provided with an incubator [8].

A series of fluorescent materials were described and proposed as samples in the ORAC test. Initially, the protein isolated from *Porphyridium cruentum*, β-phycoerythrin, was used as the fluorescent probe, which reacts with ROO^•^ to form a non-fluorescent product. However, the use of β-phycoerythrin in antioxidant assays has shortcomings for some reasons:

- β-phycoerythrin has large variability in reactivity to ROO^•^, leading to inconsistency in assay results;
- β-phycoerythrin becomes photobleached after exposure to excitation light interaction with polyphenols by nonspecific protein binding [12].

Alternative synthetic protein-devoid samples were identified as β-phycoerythrin replacements, among which fluorescein is the fluorescent sample most commonly used for the ORAC test in recent decades. Though, it was shown that fluorescein undergoes undesired fluorescence loss and secondary reactions [13], and new fluorescent molecules were proposed. Nile blue phosphorus was used as an alternative sample to determine the ORAC values in fruit juices and wines, and the values obtained were in agreement with the results of the method using fluorescein [14].

Guclu et al. (2014) described ORAC values for some liver and kidney samples measured by means of a fluorescent sample, namely p-aminobenzoic acid (PABA). The authors proposed replacing the fluorescent probe, which was normally fluorescein, with PABA in the ORAC test used for aminoacids, albumin, plasma and for some antioxidants with thiol grouping. Inconclusive results were described for thiol compounds, like glutathion and cystein [15].

Nkhili et al. (2011) stated that it is necessary to continue altering the ORAC method to remove the influence of the metallic ions in the testing systems on the ORAC values measured for the antioxidant compounds. These may lead to the formation of complex combinations with the metallic ions, which results in underestimating their ability to neutralise peroxyl radicals. Ethylenediaminetetraacetic acid (EDTA) may be useful to attenuate interferences and in this respect significantly higher ORAC values were reported when using EDTA [16].

In conclusion, this assay measures the oxidative degradation of the fluorescent, β-cyclodextrin or fluorescein molecule, after free radical generators, e.g., azo-initiator compounds, have been added. Azo-initiators produce ROO by heating, which harms the fluorescent molecule, leading to the loss of fluorescence. As oxidative degeneration continues, the fluorescent intensity decreases, and this intensity is recorded most often for half an hour after the addition of the azo-initiator as a free radical generator. Antioxidants protect the fluorescent molecule from oxidative degeneration. The degree of protection is quantified using a fluorometer. Currently, fluorescein is most often used as a fluorescent probe. Measuring equipment that can automatically calculate capacity is commercially available.

#### 4.1.2. The HORAC Test

This method evaluates the protection ability against the formation of the hydroxyl radical by means a Co(II) complex. Fluorescein is incubated with the sample to analyse, then the Fenton mixture (generator of hydroxyl radicals) is added. The original fluorescence is measured, then the readings are performed every minute after stirring. Gallic acid standard solutions with different concentrations are used to build the calibration curve. The Hydroxyl Radical Antioxidant Capacity (HORAC) test provides a direct measurement of antioxidant capacity against hydroxyl radicals by interrupting the radical reaction [17].

#### 4.1.3. The TRAP Test

**Figure 4:**
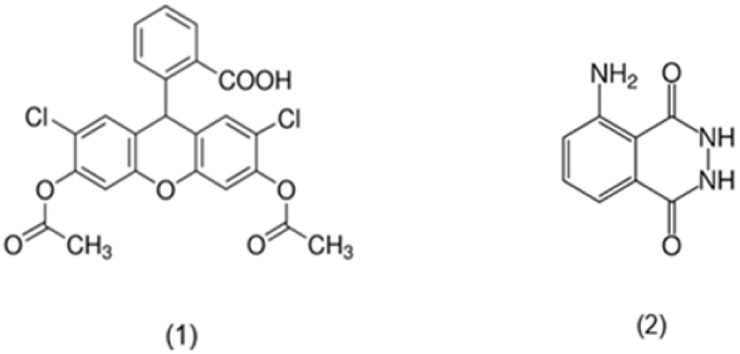
(**1**) Dihydrofluorescein diacetate; (**2**) Luminol.

The TRAP test is based on the antioxidants’ capacity to inhibit the reaction between peroxyl radicals and a target molecule, which initially represented the O_2_ consumption (as a sample) in the peroxidation process triggered by the thermal decomposition of 2,2′ azobis(2-amidinopropane)dihydrochloride (ABAP). The retardation time of the O_2_ absorption, i.e., the induction period, may be quantitatively measured and used to express the total antioxidant capacity of the samples as the TRAP value [9]. Since then, this method has been modified several times using a wider range of samples, initiators and measurements of the final point, for instance the enzymes AAPH and peroxidase were used as initiators, and fluorescein, dihydrofluorescein diacetate (DCFH-DA) and luminol as measurements of the final points of the reactions [18]. The chemical structure of dihydrofluorescein diacetate and luminol are presented in Figure 4.

The TRAP index was defined as the number of mols of ROO^•^ contained per litre of fluid (plasma) [19], according to the equation:

(1) $$\mathrm{TRAP}=R_{{\mathrm{ROO}}^{•}}\;\times \;\tau_{\mathrm{plasma}}$$

where R_ROO_^•^ is the ROO^•^ formation rate, and τ_plasma_ is the delay time in the oxygen consumption generated by the presence of human plasma.

Various methods were proposed to assess the TRAP index for natural products. One of them uses luminol (o-aminofthalhydrazide) and pyranin (8-hydroxy-1,3,6–pyrene trisulfonic acid) as target molecules. Luminol reacts with ROO^•^, emitting photons that can be measured by a luminometer, while pyranin oxidation, triggered by ROO^•^, may be traced by fluorescence measurements [9]. It can be seen that the intensity of the light emission during the incubation of luminol with AAPH is directly linked to the stable state concentration of the ROO^•^ generated in the AAPH thermolysis [9]. In the presence of the phenolic compound (or their complex mixtures), it can be seen that the stable state concentration of ROO^•^ decreases, according to the following equation:

(2) $${\left[{\mathrm{ROO}}^{•}\right]}_{\mathrm{ss}}=\frac{R_{{\mathrm{ROO}}^{•}}}{\Sigma \mathrm{ki}}$$

where [ROO^•^]_ss_ is the stable state concentration of ROO^•^, R_ROO_^•^ is the formation rate of ROO^•^, and Σki is the rate constant of all the reactions between the phenolic compound and ROO^•^.

When luminol is used as a target molecule, at high concentrations of the sample under study (high concentrations of phenolic compound), the TRAP values may be estimated.

(3) $$\mathrm{TRAP}\;=\;\frac{\Sigma \mathrm{ni}\left[X_{i}\right]}{{\Sigma n}_{\mathrm{TROLOX}}}$$

where ∑ni is the number of free radicals captured by each i molecule, [X_i_] is the micromolar concentration of this component, ∑n_TROLOX_ is the TROLOX equivalent.

Therefore, the TRAP index is directly associated to the stoichiometry of the reaction between the phenolic compound and ROO^•^ (*n*), which is defined as the number of ROO^•^ molecules captured per molecule by the phenolic compound. In any case, at low concentrations of the sample, when the concentration of the phenolic compound is insufficient for the complete protection of luminol, it may be observed that only the [ROO^•^]_ss_ decreases, without the presence of retardation times in the kinetic profiles. Under these circumstances, the TAR index may be estimated, as defined by the following equation, reflecting the reactivity of the phenolic compound towards ROO^•^.

(4) $$\mathrm{TAR}\;=\;\frac{\Sigma \mathrm{ki}}{k_{\mathrm{TROLOX}}}$$

where ∑ki is the ratio between the efficiency of the compound considered and TROLOX in decreasing stable state concentrations of the radicals involved in the process.

Depending on the potential applications of the sample studied, either the TRAP or TAR indexes may be used. If the phenolic compound is added in a certain beverage (or oil) to improve its stability, it is important to know the TRAP value of the phenolic compound included in the sample. However, if this compound will act as a radical capturer in biological systems, its reactivity to ROO^•^ (TAR) should be taken into consideration.

#### 4.1.4. The TOSC Test

The TOSC test is based on inhibiting the formation of ethylene (a control reaction is monitored by gas chromatography) in the presence of antioxidant compounds that compete with α-keto-γ-bethiolbutiric acid (KMBA) for ROS. This test uses the area under the ethylene concentration curve in comparison to the reaction time (up to 300 min) [20].

### 4.2. Tests Based on Single Electron Transfer (SET)

The tests based on the transfer of a single electron, also called electron transfer (ET) tests, detect the ability of an antioxidant to transfer an electron in order to reduce metallic ions, carbonyl groups and free radicals [21]. The SET mechanisms of antioxidant action may be summarised by the following reactions:

ROO^•^ + AH/ArOH → ROO^−^ + AH^•+^/ArOH^•+^

AH^•+^/ArOH^•+^ + H_2_O ↔ A^•^/ArO^•^ + H_3_O^+^

ROO^−^ + H_3_O^+^ ↔ ROOH + H_2_O

Relative reactivity in SET methods is based primarily on the deprotonation and ionisation potential of the reactive functional group. Therefore, SET reactions are pH dependent [22]. The aryloxyl radical (ArO^•^) is subsequently oxidised to the corresponding quinone (Ar = O). The more stabilised the aryloxyl radical, the easier the oxidation from ArOH to Ar = O due to the reduced redox potential. The antioxidant action in these tests is often simulated with a suitable fluorescent or coloured sample instead of peroxyl radicals.

The spectroscopic SET tests, including the Folin–Ciocalteu test (FC), the ferric reduction of antioxidant power (FRAP) and the tests of reducing the cupric antioxidant capacity (CUPRAC), measure the capacity of an antioxidant to reduce an oxidant, which changes colour when reduced. The colour change degree is correlated with the concentration of the total antioxidant capacity. Moreover, electrochemical and nanotechnological methods also belong to the category of ST-based tests.

#### 4.2.1. The CUPRAC Test

The CUPRAC assay for determining the total antioxidant capacity was devised in the early 2000s [23], but it has already been modified for various methods of measuring the antioxidant activity based on the reduction of cupric (Cu^2+^) to cuprous (Cu^+^). Similar to other assays, a ligand is employed to form a copper–ligand complex to facilitate absorbance measurement. Neocuproine (Nc; 2,9-dimethyl-1,10-phenanthroline) is the ligand commonly employed in CUPRAC assay. This method has been applied to various matrices containing both lipophilic and hydrophilic antioxidants.

**Figure 5:**
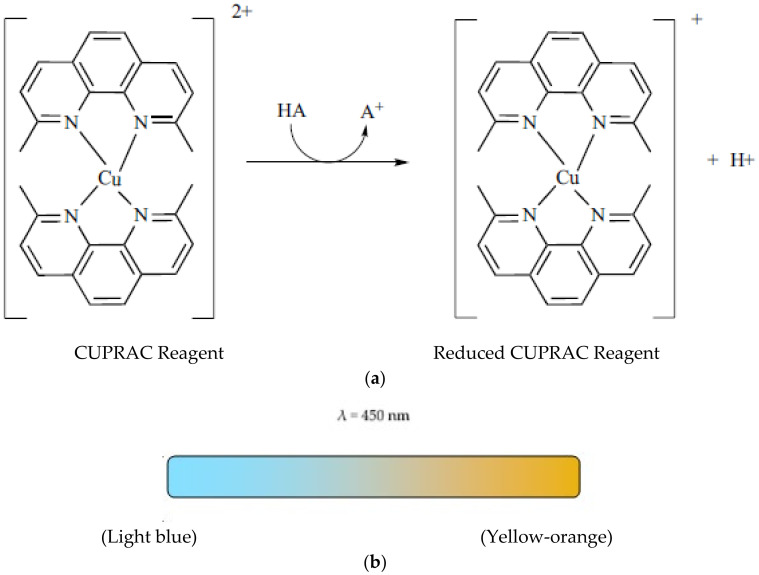
Reaction scheme involved in Cupric Reducing Antioxidant Power (CUPRAC) assay. HA represents an antioxidant molecule and A^+^ an oxidised antioxidant molecule (**a**); Colour change in the assay (**b**).

This method can be used for the determination of the antioxidant capacity of food constituent by the Cu^2+^–neocuproine reagent as the chromogenic oxidising agent. The reduction of Cu^2+^ in the presence of neocuproine by a reducing agent yields a Cu^+^ complex with maximum absorption peak at 450 nm (Figure 5). The chromogenic oxidant reagent of the CUPRAC assay, Cu^2+^–Nc, reacts with the n-electron reducing antioxidant substances (AOX), as shown in the next equation:

*n*Cu(Nc)_2_^2+^ + *n*-electron reductant (AOX) ⇄ *n*Cu(Nc)_2_^+^ + *n*-electron oxidized product + *n*H^+^

In this reducing assay, the reactive Ar-OH groups of polyphenols and other antioxidants are oxidised to the corresponding quinones and Cu^2+^–neocuproine is reduced to the Cu^+^–neocuproine complex, which is intensely coloured in yellow–orange. It should be noted that the real oxidant is the Cu(Nc)_2_^2+^ species and not Cu^2+^ alone, since the standard redox potential of the Couple (II/I)-Nc is 0.6 V, much higher than that of the noncomplexed couple Cu^2+^/Cu^+^ (0.17 V) [9]. The main antioxidants in foodstuffs and biological compounds have a redox potential corresponding to the range of 0.2–0.6 V, according to that of the redox couple Cu(II/I)-Nc.

Taking into account the favourable attachment of the cupric state with neocuproin, the quantity of chromophore product occurring at the end of the reaction equals that of Cu (II)-Nc. The released protons are buffered in a solution containing ammonium acetate, with pH of 7.0. This pH is optimum for CUPRPAC assay because it is very close to the physiological pH (7.4).

For some specific compounds, including epicatechin galate, rosmarinic acid, quercetine, epigalocatechin, catechin, acid caffeic acid, epicatechin, gallic, rutin, and chlorogenic acid [24,25], the best antioxidant capacities were noted using the CUPRAC method. This was possible because an optimal transfer of the electrons depends on the number and orientation of the hydroxyl groups and, also, on the degree of conjugation of the entire molecule [26].

Despite the fact that there are other derivatives of phenantrolin that may selectively stabilise the Cu^+^ ion in relation to the Cu^2+^ ion, among which are BCS (2,9-dimethyl-4,7-diphenyl-1,10-phenanthroline disulfonic acid) [27] and bicinconinic acid (BCA: 2-(4-carboxyquinolin-2-il) chinolin-4-carboxylic acid) [28], only neocuproin as a primary ligand for the CUPRAC assay is widely used in measuring antioxidant capacity. It is worth mentioning that BCS and BCA have particular disadvantages in comparison to Nc. First of all, due to the presence of negatively charged sulfonate groups on the fenantrolin ring, the Cu(I)–BCS complex has a higher global charge than the Cu(I)–Nc complex. Ion–dipol interactions with water molecules cause reverse addiction—proportional between the charge on the chromophore and its affinity for hydrophobic expulsion. Therefore, Cu(I)–BCS will unavoidably have a lower permeability of the membrane, which is why it will be less frequently used as a TAC reagent in nonpolar solvents compared to copper–Nc.

In terms of reaction kinetics and the answer to the antioxidant compounds of the lipophilic plasma (for instance, β-carotene, α-tocopherol), from Çelik et al. (2012) point of view, the Cu(I)–BCS test cannot be compared practically with CUPRAC assay [29].

Zhou et al. (2012) showed that the reduction potential for the Cu(I,II)–BCS couple had the value of: E° = 0.844 V [30], slightly higher than the ones of the most common ET reagents. This can negatively influence selectivity for genuine antioxidant substances.

On the other hand, even if BCA has a higher wavelength of maximum absorption, which is apparently advantageous (558 nm), as compared to Nc for its cupric complex (which allows the removal of the background colour from most plant pigments), it was noticed that, while conducting the BCA test, the concentration of free cupric ions cannot be preserved in excess [31].

Moreover, it was reported that the results obtained from in vitro cupric ion (Cu^2+^) reducing measurements might be more efficiently extended to the possible in vivo reactions of antioxidants. CUPRAC chromogenic redox reaction is carried out at a pH (7.0) close to the physiological pH, which has a value of 7.4. Reaction time to reach completion may vary between 30 and 60 min, depending on how fast the antioxidant is. For instance, flavonoid glycosides may require preliminary hydrolysis to fully highlight their antioxidant capacity.

The original CUPRAC test was modified to include various samples in diverse applications. For example, the acetone/water environment, with the help of methyl-β-cyclodextrine, was used to simultaneously determine hydrophilic and lipophilic antioxidants [32]. In order to evaluate phenolic antioxidants in free forms, as well as in tied forms in the food matrix without preliminary extraction and reducing the intricate hydrolysis process, a proposition was made to forcefully solubilise the antioxidants attached to the CUPRAC reagent, which has advantages as a result of the phenomenon of superficial reaction between the solid material (tied antioxidants) and the liquid material (soluble CUPRAC reagent) [33]. This modified method was suggested as applicable to relatively insoluble food matrices, as well as insoluble cosmetic products, such as creams, balms and powders.

In another modified CUPRAC test, an optical sensor was modified to contain immobilised chromogenic redox reagent to measure the reduction power of liquid samples without any pretreatment [25]. The CUPRAC Cu^2+^–neocuproin reagent was immobilised on a cation-changing Nafion film, and the complex was reduced by antioxidants to Cu^+^–neocuproin, yielding modifications of absorbance at 450 nm, which were monitored spectrophotometrically. Another version of the optical sensor aiding the CUPRAC test used a miniaturised reflection spectrophotometer to measure the changes of reflection at 530 nm instead of absorbance [34].

The use of the optical sensor was suggested so to considerably simplify operation, as it is reliable and robust, and therefore able to allow for the in situ estimation of the antioxidant capacity of the various food extracts and biological samples.

The CUPRAC test, together with its adjustments for the measurements aimed at eliminating reactive species of oxygen, was analysed and certain advantages were highlighted in comparison to other ET-based assays, in an extensive analysis performed by Özyürek et al. (2011) [35].

- The CUPRAC reagent is fast enough to oxidise thiol-type antioxidants, while other ET assays based on Fe(III), such as the FRAP method, do not allow the measurement of certain tiol antioxidants, such as glutathione. The reason for this may be the electronic structure of Cu(II) that enables fast kinetics.
- Favourable pH: the redox reaction producing coloured species is carried out at pH 7.0 buffer as opposed to the acidic conditions (pH 3.6) of FRAP, or basic conditions (pH 10.0) of the Folin–Ciocalteu assay. At more acidic conditions than the value of 7.4 corresponding to the physiological pH, the reducing ability may be suppressed. The reason for this is the protonation on antioxidant compounds while, in more basic conditions, acid dissociation (through proton release) from phenols improves the reduction capacity of a sample, thus triggering unrealistic measurements of TAC.
- Favourable redox potential: the CUPRAC reagent is selective, because it has a lower redox potential than that of the ferric–ferrous couple in the presence of phenanthroline—or similar ligands. The standard potential of the Cu(II,I)–Nc redox couple is about 0.6V, close to that of ABTS^+^/ABTS, i.e., 0.68 V. Due to the fact that simple sugars and citric acid are not true antioxidants, these compounds cannot be oxidised with the CUPRAC reagent.
- Stability and facility: the reagent is more stable and accessible than other chromogenic reagents (e.g., ABTS, DPPH).
- Versatility: this method is capable of measuring both hydrophilic and lipophilic antioxidants (e.g., β-carotene and α-tocopherol). The lipophilic antioxidants of serum may be assayed separately from the hydrophilic ones by hexane extraction of serum, followed by colour development in dichloromethane. As an advantage over the widely used Folin–Ciocalteu reagent, CUPRAC can measure lipophilic antioxidants, while FCR cannot be used for TAC assay of biological fluids.
- According to a comprehensive review compiled by Christodouleas et al. (2014), lipophilic antioxidant substances in edible oils may be efficiently analysed in order to obtain the TAC values by means of the Cu(II)–Nc reagent [36].
- Robustness: it is observed that physiological parameters, such as air, humidity, sunlight do not influence the CUPRAC reaction with antioxidants, as opposed to the reagents of the free radical type, such as DPPH.
- Sensitivity and linear response: the absorbance vs. concentration curves are linear in the CUPRAC method over a wide range, unlike those of other methods yielding polynomial curves. The molar absorptivity, i.e., (7.5–9.5 × 10^3^ *n*) Lmol^−1^cm^−1^ for n-electron reductants, is sufficiently high to sensitively determine biologically important antioxidants.
- The TAC values of antioxidants found with CUPRAC are perfectly additive, i.e., the TAC of a mixture is equal to the sum of TAC values of its constituents. Unfortunately, because of the fact that the oxidation reactions cannot be controlled, the additive property of antioxidant capacities cannot be ensured for complex mixtures in various similar TAC tests (for example, thiol compounds showed a lack of additivity with a number of polyphenol compounds in FRAP responses) [29].
- Absence of prooxidative behaviour: since the Cu(I) ion emerging as a product of the CUPRAC redox reaction is in chelated state (i.e., Cu(I)–Nc), it cannot act as a prooxidant that may cause oxidative damage to biological macromolecules in body fluids. The stable Cu(I)–chelate does not react with hydrogen peroxide, but the reverse reaction, i.e., oxidation of H_2_O_2_ with Cu(II)–Nc, is possible. Thus, Cu(I) chelated to Nc may not act as a prooxidant toward the tested antioxidants in a Fenton-type reaction in the absence of H_2_O_2_ or its precursors.
- The method is easily and diversely applicable in conventional laboratories using standard colorimeters rather than necessitating sophisticated equipment and qualified operators [37].
- Applicability in foods and medicine: according to the research teams coordinated by Gorinstein et al. (2006), the CUPRAC test showed repeatable results in point of real pH and the redox potential for certain diversified food extracts, among which kiwi, garlic, and onion [38]. Bean and his team (2009) described a comparative analysis on antioxidant reactivity within the obstruction and control of bladder tissue in rabbits, involving two different TAC tests. The CUPRAC test, unlike the FRAP test, detected a considerable decrease in the antioxidants found in the obstructed bladder tissue as compared to the control bladder tissue in both the muscles, and the mucous membrane. Bean et al. (2009) concluded that the CUPRAC test was receptive to hydrophilic, lipophilic antioxidants, and antioxidants containing thiol at physiological pH, and as a result, it was a much better tool for the analysis of antioxidant reactivity in tissues [39].

#### 4.2.2. The FRAP Test

The FRAP test is a typical SET-based method measuring the reduction of the complex of ferric ions (Fe^3+^)-ligand to the intensely blue ferrous complex (Fe^2+^) by means of antioxidants in acid environments. Antioxidant activity is determined as an increase in absorbance at 593 nm, and the results are expressed as micromolar equivalents of Fe^2+^ or in relation to a standard antioxidant [5]. Unlike other SET-based methods, the FRAP test is carried out in acidic pH conditions (pH = 3.6)to maintain iron solubility. Reaction at low pH decreases the ionisation potential that drives electron transfer and increases the redox potential, causing a shift in the dominant reaction mechanism [40].

The original FRAP test uses tripyridyltriazine (TPTZ) as the linking ligand to the iron ion, while alternative ligands were also used to bind the iron ion, such as ferrozine to assess the reducing power of ascorbic acid [41].

More recently, potassium ferricyanide has been the most common ferric reagent used in FRAP tests. In this latter case, Prussian blue is obtained as a final product that may be quantified spectrophotometrically and shows the reducing power of the antioxidants tested. The formation of Prussian blue may occur in two different manners with the same result. Antioxidants can either reduce the Fe^3^^+^ in the solution to Fe^2^^+^, which binds the ferricyanide to yield Prussian blue, or reduce the ferricyanide to ferrocyanide, which binds the free Fe^3^^+^ in the solution and forms Prussian blue.

**Figure 6:**
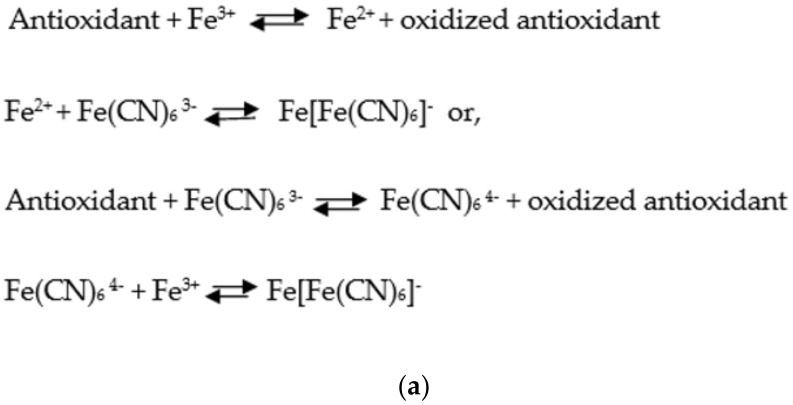

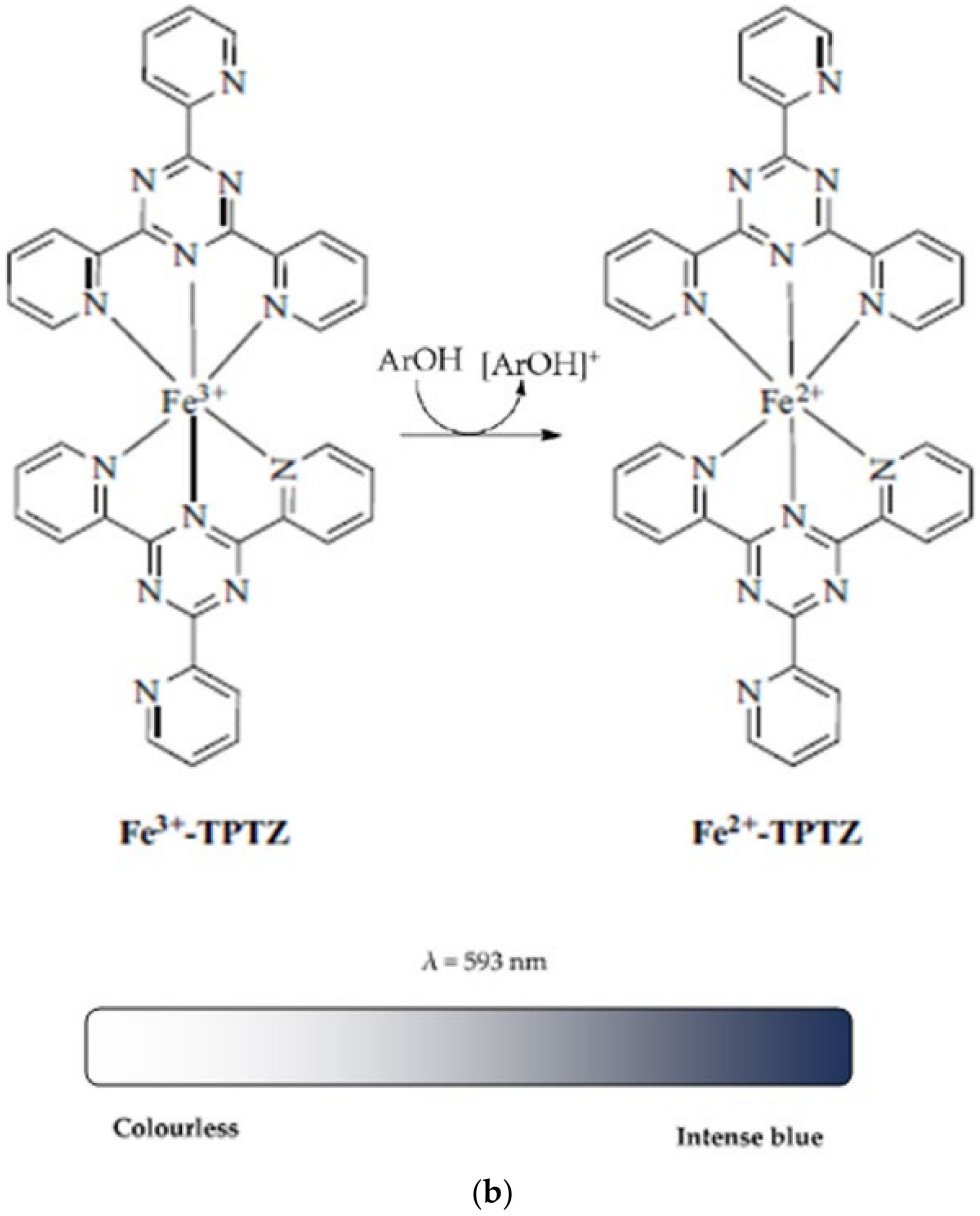
Ferric reducing antioxidant power (FRAP) reaction mechanism. ArOH represents a phenolic antioxidant and [ArOH]^+^ an oxidised phenolic antioxidant (**a**); Chemical structure of the complexes involved in the chemical reaction and the colour change (**b**).

Figure 6 shows the simplified scheme for these two reactions (a) and the colour change together with the mechanism of reaction (b) [42]:

A disadvantage of this FRAP test is the tendency of Prussian blue to precipitate, to form a suspension and stain the measurement vat. Therefore, the time to add Fe^3+^ (FeCl_3_) is essential and may produce errors in the interpretation of the results. In order to stabilise Prussian blue against precipitants, Berker et al. (2010) proposed adding sodium dodecyl sulphate, a tensioactive compound, and an optimal pH in order to maintain the redox activity of the ferric ion and prevent the hydrolysis process [42]. The authors suggested that this modification also allows the evaluation of the antioxidants, whose redox potential does not exceed the one of Fe^3+^/Fe^2+^ in the conventional FRAP test, such as thiol-type physiological antioxidants. The FRAP test was further modified by selecting water/acetone as solvent in the absence of the randomly methylated β-cyclodextrine (RMCD) solubility potentiator to allow the simultaneous measurement of hydrophilic and lipophilic antioxidants, which was restricted in the conventional FRAP test [43].

The FRAP test is simple, fast and cost-effective, and does not require specialised equipment. However, Pulido, Bravo and Saura-Calixto (2000) reported that the FRAP results may vary according to the observed analysis time for the reaction between the antioxidants and Fe^3+^, which ranged from a few minutes to several hours [44]. Therefore, a single-point absorption endpoint may not represent a complete reaction, since various antioxidants require different reaction times for detection [8].

The FRAP test has recently adopted electrochemical detection techniques for better sensitivity, accuracy and reproducibility. A new method of coulometric titration was developed, to determine the FRAP value of different antioxidant materials [45]. In this modified FRAP method, the antioxidant reacts with the coulometric titrants (electrogenerated ferricyanide ions), and the amount of electrical energy consumed for titration up to the final point (when the initial current is resumed) serves as an indicator for the reduction of the antioxidant power [46]. The FRAP test by coulometric titration stands out by being extremely sensitive, reliable and simple in assessing reductive power. Another electrochemical version of the FRAP test is the chronoamperometric measurement of the antioxidant’s reducing power. As proved by Brainina, Varzakova and Gerasimova (2012), a change in the potential of the K_3_[Fe(CN)_6_]/K_4_[Fe(CN)_6_] system is indirectly correlated to the reducing power of the antioxidant present. More precisely, at certain potentials (higher than 0.35 V), the oxidation process takes place only forming K_3_[Fe(CN)_6_], and the concentration of K_4_[Fe(CN)_6_] is entirely from the reaction of K_3_[Fe(CN)_6_] with the antioxidant, being proportional to the antioxidant’s reducing power [47]. This chronoamperometric method was proposed to assess the antioxidant activity of biological samples due to its good detection limit (e.g., 2 × 10^−6^ M for ascorbic acid).

Generally, the FRAP test, as a non-radical SET-based method, was promoted as having a low relation with the process of radical extinction (the HAT mechanism) occurring in lipid systems and has a low correlation with other antioxidant activity measurements. As a result, it is suggested that this test may be used together with other methods to distinguish the dominant mechanisms for different antioxidants [8].

Ascorbic acid (vitamin C) is an important physiological antioxidant [48]. Many plants are rich in vitamin C and are important dietary agents, as people must ingest vitamin C, but cannot synthesise or store it. A simple adjustment of the FRAP test allows the measurement of the ascorbic acid in the same sample and in the same manner as the FRAP test [49]. The modified test is known as the analysis of the reducing ferric and ascorbic acid antioxidant power, called FRASC, and was validated in comparison to a reference HPLC method [50,51].

The concept of redox reducing capacity as an index of antioxidant activity may be applied in different manners. There are several tests based on transitional metals (iron and copper), like those using ferricyanide, ferrozine, Prussian blue or cupric ions instead of Fe–TPTZ [52]. In addition, the methods which were developed use changes of the redox electrochemical signals [9]. However, the FRAP test is the most common; it is well validated and has generated large amounts of data on foods, beverages, body fluids and other types of samples. Various modifications were introduced by various users. These are usually small, involving modifications of pH, temperature, calibration method, reaction duration and reporting units. However, the variations that occur make it difficult to compare the results of different studies. Thus, except for the existence of an imperative reason for changing the reaction conditions for an experimental series or a certain sample type, the FRAP test should use a standard operating procedure, so that the results may be interpreted in association with the data published using the same method for the index of total antioxidant activity (NEAC). If modifications are operated at the level of the reaction conditions or duration, standardisation or the manner of expressing the results, then it is important that they should be justified and clearly described, and the modified method should be validated in comparison to the standard procedure.

The FRAP test is used globally on a large scale, providing results for a variety of purposes, including the estimation of the antioxidant content in foods and their contribution to the supply of antioxidants, to investigate the effect of storage, growth, draught, solar radiation, processing, genetic modification of dietary agents and petfoods, and to compare the relative content of antioxidants in foods, medicines, traditional medicines, herbs, spices, teas and wines for product differentiation, quality, control and development.

The FRAP test may be also used to detect water contamination, and was used to study the effect of radiations, pollution, climate change and space travel on living organisms. The high sensitivity and accuracy of the test allow discrimination among samples, which was used in assessing antioxidant absorption and systemic distribution (“bioavailability”) after ingesting foods, beverages, medicines or supplements.

F.T. Pastor et al. (2020) examined the applicability of the reduction reaction of Fe(III) to Fe(II) by the antioxidants in order to develop the electrochemical method of determining antioxidant activity, applying direct current polarography and cyclic voltammetry. The redox Fe(III)/Fe(II) system was chosen not only because it is the basis for the simplest spectrophotometric test for antioxidant activity measurement, but also because it is the standard reversible redox system in chemistry. Developed in a fast and simple manner, and lacking the need for calibration according to a standard (like Trolox or gallic acid), electrochemical methods were applied to measure the antioxidant activity for ten natural antioxidants. The results obtained were compared to the antioxidant activity of the same antioxidants, measured by means of the FRAP, ABTS and DPPH tests [53].

Furthermore, the FRAP test was widely used to investigate the effects of lifestyle, diet, supplements, pregnancy, age, gender, disease, medical treatment, traditional/alternative therapies on the total antioxidant activity of biological fluids, including plasma, urine, saliva, follicular fluid, alveolar liquid, seminal material, tears, cerebrospinal fluid, and faeces. In the biomonitoring and supplementary studies, the FRAP test was used in human investigations, as well as on samples from various animals, insects, and marine organisms.

#### 4.2.3. The Folin–Ciocalteu Method

The Folin–Ciocalteu test is a well-known method aimed at determining the total phenolic content (TPC). The Folin-Ciocalteu test was widely used in clinical and nutritional studies to measure the total polyphenolic content in plant-derived foods and biological samples. This method was originally designed to analyse proteins, but it was later adopted by Singleton, Orthofer and Lamuela-Raventos (1999) in order to analyse the phenolic components in wine, after which it became a routine test for the antioxidant evaluation of food and plant extracts [54].

At present, the Folin–Ciocalteu test is commercially available from several important commercial societies, so this method is widely used to quantify polyphenols in plant-derived extracts [55], as well as in foods and beverages [9]. The pharmacopoeia includes the Folin–Ciocalteu test [9], and Europe adopted it as an official procedure of measuring the total phenol contents in wines (European Community 1990).

**Figure 7:**
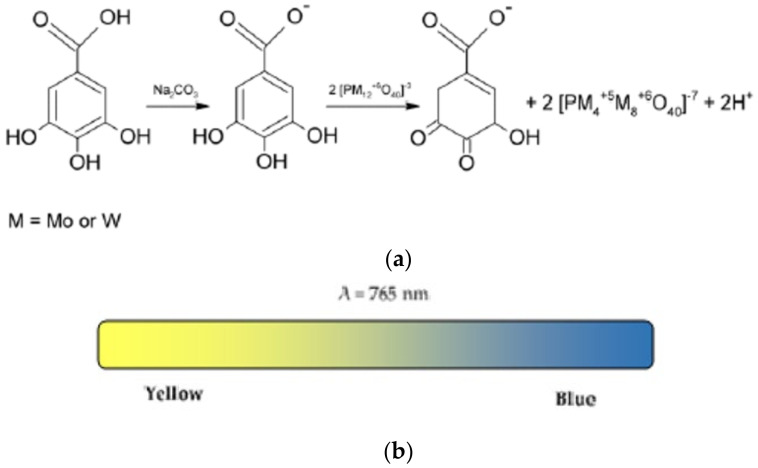
Reaction between the phenolic compounds and the derivatives of the phosphotungstic and phosphomolybdic acids in an alkaline environment, resulting in the formation of a blue colour by the Folin–Ciocalteu method (**a**); Colour variation observed in the assay (**b**).

**Figure 8:**
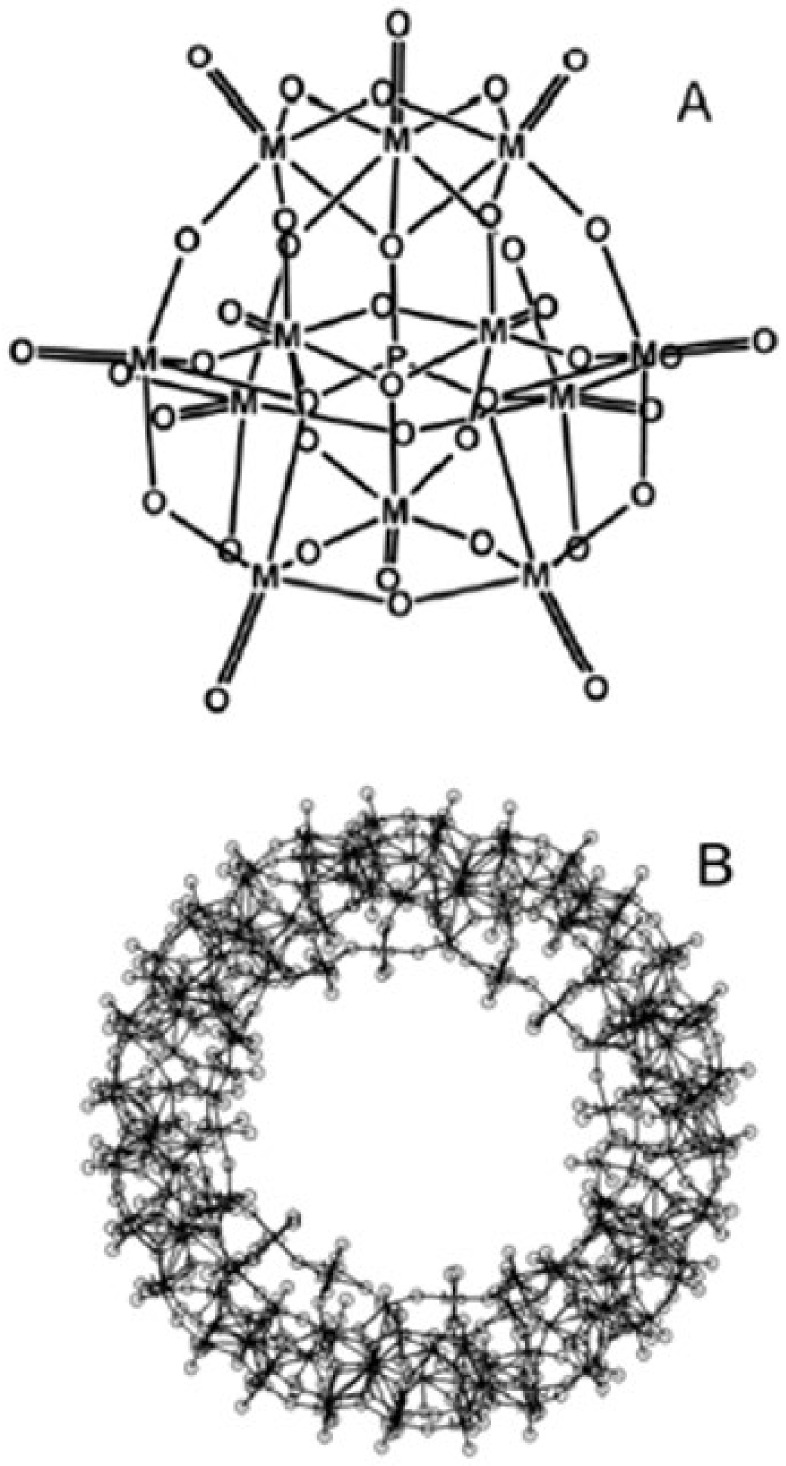
(**A**) The α-Keggin structure of the anionic derivative [PM_12_O_40_]^3−^, where M stands for molybdenum (Mo) or tungsten (W); (**B**) the big wheel structure of the blue complex [Mo_126_^6+^Mo_28_^5+^O_462_H_14_(H_2_O)_70_]^14−^.

The Folin–Ciocalteu test is based on reducing the Folin–Ciocalteu reagent with phenolic compounds in an alkaline state. The exact chemical nature of the Folin–Ciocalteu reagent is not clearly defined, but it is believed that it may contain a complex of the phosphomolybdic/phosphotungstic acid which are reduced to obtain a blue chromophore with the maximum absorption at 765 nm (Figure 7) [56]. The central molybdenum ion in the complex is accepted as a reducing site, where the Mo^6+^ ion is reduced to Mo^5+^ by accepting an electron donated by the phenolic antioxidant. The anionic derivatives of the phosphotungstic and phosphomolybdic acids have an α-Keggin structure and the blue complex has a big wheel structure Mo154—of the cluster-type (Figure 8) [57].

Thus, the Folin–Ciocalteu test is an SET-based test, and it is associated to the reducing power of phenolic antioxidants. Gallic acid is the commonly used reference standard, and the TPC results are usually expressed as Gallic acid equivalent. However, the TPC results are also occasionally expressed as catechins, caffeic acid, chlorogenic acid or the equivalent of ferrulic acid, requiring the standardisation of the reported results [58].

The Folin–Ciocalteu assay for TPC measurement has numerous advantages, including simpleness, reproducibility and robustness. However, it also has some drawbacks. First of all, the test is sensitive to pH, temperature and reaction time, and that is why it is necessary to accurately select the reaction state for coherent and reliable results. Secondly, TPC overestimation is a major concern for the Folin–Ciocalteu test, owing to the contribution of the non-phenolic reducing agents present in the system when reducing the Folin–Ciocalteu reagent [59]. Such examples of contaminants include reducing sugars and certain amino acids. Thus, the results of TPC measurements may be overestimated by one size for comparison to the ones obtained by HPLC methods. Additionally, the test is performed in aqueous systems, and its application for lipophilic phenols is limited, except for the case when modifications of the solvent system are applied.

To improve the durability and reduce the cost and time of the Folin–Ciocalteu test, a methodology for microtitre 96 plates was devised for food samples [60] and urine samples [61].

The level of polyphenols in urine measured by the Folin-Ciocalteu test was used as a biomarker of the polyphenol supply and correlated to a decrease in the cardiovascular risk parameters, such as high blood pressure, an increase in the nitric oxide as a potentially relaxing agent [62], improvements of the lipid profile and the glucose response [63] and a decrease in DNA oxidation. Another advantage was improved cognitive performance, which was associated to an increased supply of polyphenol-rich foods [64].

An evaluation of the antioxidant capacity of polyphenols according to their chemical structures was carried out by B. Alcalde et al. (2019). Several tests were employed, among which were Folin–Ciocalteu (FC), FRAP and TEAC to select the polyphenols differing in number and position of the hydroxyl groups. Voltammetric methods with screen-printed carbon electrodes were also recorded within the range −0.2 to 0.9 V (vs. the Ag/AgCl reference electrode) to investigate the oxidation behaviour of these substances. Weak correlations among tests were obtained, which means that each compound had a behaviour that varied as a response to the different methods used [65].

### 4.3. Mixed Mode Tests (HAT/SET)

These mixed mode tests are generally based on the elimination of a stable chromophore (like 2,2′-azinobis-3-ethylbenzthyazolin-6-sulfonic acid) (ABTS) and 2,2-diphenyl-1-picrylhydrazil (DPPH)), where HAT, ET, and proton-coupled electron transfer (PCET) mechanisms may play different roles in varied proportions, depending on the corresponding reaction conditions (such as pH and solvent) [66]. Mixed mode tests (HAT/SET) mainly include the ABTS/Trolox equivalent antioxidant capacity (TEAC) test, the DPPH radical neutralisation test, and the N,N-dimethyl-p-phenylenediamine dihydrochloride (DMPD) radical neutralisation test.

#### 4.3.1. The ABTS (TEAC) Test

**Figure 9:**
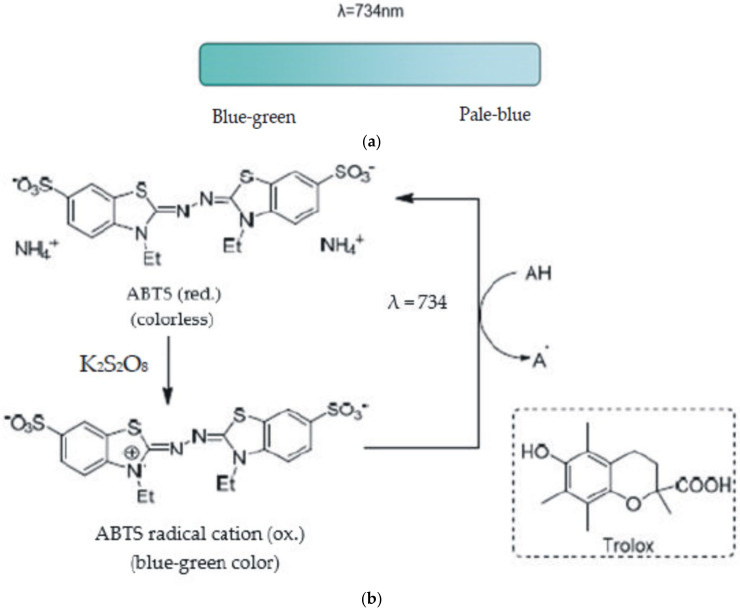
Colour variation in ABTS assay (**a**); Reaction scheme involved in 2,2′-Azinobis-(3-ethylbenzothiazoline-6-sulfonic acid (ABTS) radical cation scavenging activity assay (**b**).

The TEAC test was first developed by Miller and his team (1993) as a simple and convenient method used to measure the total antioxidant capacity (TAC) [67]. the test measures the antioxidants’ capacity to neutralise the 2,2′-azinobis(3- ethylbenzthiazolin-6-sulfonic acid) (ABTS^•+^) stable radical cation, a blue-green chromophore of maximum absorption at 734 nm, whose intensity decreases in the presence of antioxidants. ABTS^•+^ may be generated from ABTS in the presence of powerful antioxidant agents. The degree of discolouration of the blue–green colour, quantified as a sudden drop in absorbance to 734 nm, depends on the reaction duration, intrinsic antioxidant activity, and sample concentration (Figure 9).

In the original TEAC test, metmyoglobin and hydrogen peroxide are used to generate an intermediate radical of ferrylmyoglobin, which subsequently reacts with ABTS to produce ABTS^•+^. Later on, the oxidant agent was replaced with peroxide or persulphate. Potassium persulphate is the most common oxidant for ABTS^•+^ generation.

As can be seen in the reactions below, in the system formed by ABTS/H_2_O_2_/peroxidase, ABTS behaves as a reducing agent, substituting the enabled form of the enzyme (named compound I) in compound II, which returns to the initial form of the enzyme (E).

E (native form of peroxidase) + H_2_O_2_ → Compound I + H_2_O

Compound I + ABTS → Compound II + ABTS^•+^ (ABTS cation-radical)

Compound II + ABTS → E + ABTS^•+^

ABTS is oxidised by oxidants to its radical cation, ABTS^•+^ which is intensely coloured in blue–green. Furthermore, the ABTS radical is soluble in water and organic solvents, enabling the determination of antioxidant capacity of both hydrophilic and lipophilic compounds.

Durmaz (2012) prepared a ready-to-use ABTS^•+^ radical powder by incubating ABTS with potassium peroxodisulphate, followed by drying and freezing the resulting solution [68]. This lyophilised radical powder was then used as a replacement for the freshly prepared solution, and the linearity and accuracy of the modified test were proven by the authors.

Bleaching of a preformed solution of the blue–green radical cation ABTS^•+^ has been extensively used to evaluate the antioxidant capacity of complex mixtures to induce the oxidation reaction of aromatic alcohols, with corresponding aldehyde formation. In addition, ABTS has been used to determine p-hydroxybenzoic acids and polyphenolic compounds by means of the enzyme laccase [69,70] and, also, to determine a variety of flavonoids using peroxidase [71]. In both situations, as a result of the enzymatic reaction, an ABTS–polyphenol complex is formed.

The TEAC test was used to measure the total antioxidant capacity of pure substances, corporal fluids and vegetable materials. The TEAC test, similar to other methods of radical neutralisation, may be automated and adapted to microplates and flow injection techniques. It may also be coupled with HPLC by including a post column reaction with the ABTS radical–cation to facilitate the identification of individual antioxidants in a complex mixture. HPLC–TEAC provides a fast and effective method of separation and identification of the bioactive compounds in the source material [72]. A higher sensitivity and efficiency of the TEAC test may be achieved when the method is coupled with other detection techniques, like amperometry [73] and FTIR [74]. Many of these modified TEAC assays use the online enzymatic generation of ABTS^•+^, mainly those employing continuous flux systems. For example, Milardovic et al. (2007) reported generating ABTS^•+^ by glucosoxidase and peroxidase separately immobilised in tubular flow reactors for the analysis of the TEAC values of alcoholic beverages [75].

ABTS radical scavenging method can be evaluated over a wide pH range, which is useful to study the effect of pH on antioxidant mechanisms for food components. Furthermore, the ABTS radical is soluble in water and organic solvents, enabling the determination of antioxidant capacity of both lipophilic and hydrophilic compounds. In the case of lipophilic compounds, such as carotenoids, tocopherols, etc., homogeneous solutions were used to assess the degree and protective capacity of lipid-soluble antioxidants on lipids.

According to the protocol developed by Miller et al. [76] regarding the estimation of the antioxidant activity of carotenoids, these substances were dissolved in acetone and diluted in a mixture of hexane and acetone (90:10 v/v), using manganese dioxide as a reaction medium. Böhm et al. (2002) changed this method by using hexane as solvent for dissolving carotenoids. This dissolution stage was followed by centrifugation and measurement of the inferior blue–green hydrophilic layer’s absorbance [77]. However, these tests occasioned the occurrence of serious reactivity issues between the carotenoids and ABTS^•+^ in aqueous environment [78].

Based on the capacity of the horseradish peroxidase enzyme to operate in organic environments, Cano et al. (2000) described a method that involves the direct production of cation in such environments [79]. Various solvents were used, like methanol, ethanol, acetone and dimethylsulfoxide, subsequently establishing the kinetics of the reaction and also the stability of the radical. The time to generate the ABTS^•+^ radical by peroxidase enzyme was approximately 100 s, using ethanolic media. The shortcoming in this activity to generate the radical ABTS^•+^ has been linked to its solubility in such environments [80]. The total antioxidant activity (TAA) of both hydrophilic and lipophilic compounds has been determined by combining lipophilic antioxidant activity (LAA) and lipophilic activity (LAA) through the ABTS^•+^ radical cation.

Puangbanlang et al. (2019) reported the first use of a paper-mounted device as a simple, cheap and fast detection platform for the simultaneous determination of the antioxidant activity and the total phenolic content in food samples. Two antioxidant activity tests, including the analysis of the cation radical (ABTS) and the analysis of the reducing antioxidant capacity of the cupric ion (CUPRAC), as well as a test for the total phenolic content, the Folin–Ciocalteu test (FC), were used at the same time. The device consisted of a central sampling area connected to four consecutive pre-treatment and detection areas hosting all the three tests and a witness measurement of the sample. The test was performed by fixing the samples in the sampling area in order to flow through the pre-treatment and detection areas containing the reagents stored for each antioxidant test, triggering the colour change which was measured photocolourimetrically. Test optimisations included the concentrations of the key reagents, reaction time and surface adjustment, being performed to obtain sensitive tests, with wide linearity domains. Various antioxidant standards were subsequently evaluated in order to determine the analytic characteristics of the method. The paper-based tests were successfully applied to detect the antioxidant activity and total phenolic content in 10 beverages with Gallic acid equivalent values (GAE) similar to those obtained in traditional tests, with a confidence interval of 95%. In addition, the GAE values of the samples obtained as a result of the three analyses of the paper-based test were well correlated among themselves with relatively high Pearson correlation coefficients. These results showed that the paper-based test yielded accurate results and is suitable for the simultaneous analysis of the antioxidant activity and total phenolic content in real samples [81].

##### Advantages of the ABTS Test

- TEAC tests allow the determination of a large variety of antioxidant substances, since ABTS^•+^ radical reacts rapidly with both synthetic and natural antioxidant substances (i.e., phenols, amino acids, peptides, vitamin E and vitamin C) in food components [82].
- The TEAC antioxidant test may be used over a wide pH range, although, in many cases, the sample for which antioxidant activity is measured may influence the pH value [83]. This is due to the fact that the reaction mechanism may vary with pH, e.g., the electron transfer is facilitated by acid conditions [84].
- ABTS^•+^ solubility in buffered and organic environments led to the development of methods for the determination of hydrophilic and lipophilic antioxidant activity [80], whose sum yielded an accurate determination of the antioxidant capacity of the products [85].
- The TEAC assay is cheap and operationally simple.

##### Disadvantages of The ABTS Test

In kinetic tests, the reaction involved in the TEAC test is uncertain because the test substance can react with the oxidiser, the enzyme and the radical cation, thus obtaining an overrated value [86]. While the discolouration test may solve this problem, other disadvantages occur because of the problems of procedure and mechanism [87].

The TEAC assay has also been challenged for its lack of biological relevance due to use of the artificial ABTS radical cation that is not found in food or biological systems [88].

Many phenolic compounds have low redox potentials and, therefore, can react with ABTS^•+^. Additionally, the TEAC reaction may be different for slow reactions, and it may take a long time to reach an endpoint. In such cases, using an endpoint of short duration (4 or 6 min), may lead to underestimation of the antioxidant capacity due to reading before the reaction is finished [89].

#### 4.3.2. The DPPH (2,2-di(4-tert-octylphenyl)-1-picrylhydrazyl) Test

##### Characteristics of The DPPH Radical

DPPH^•^ is a π-radical, present in its monomer form in a solid state, as well as in solution. The first structural data showed that its unique low reactivity was mainly influenced by the “efficient screening of the hydrazyl structure by the molecule’s surrounding parts” and less by extended conjugation [90]. It was confirmed that, although the removal of the p-nitro group only has a slight influence, the removal of the group responsible for protecting the o-nitro groups led to a significant reactivity increase. Moreover, the fact that both phenyl groups are twisted has an adverse effect on conjugation stability.

The radical is soluble in different organic solvents, but not in water. It usually dissolves in methanol, ethanol, or their aqueous mixtures. In this final case, the water content should not exceed 60% to make the radical more readily soluble [91]. At a high water content, the typical quintet spectrum of the dissolved DPPH^•^ is converted to singlet, typical for a solid state radical. Such a modification of radical solubility is not always visible.

**Figure 10:**
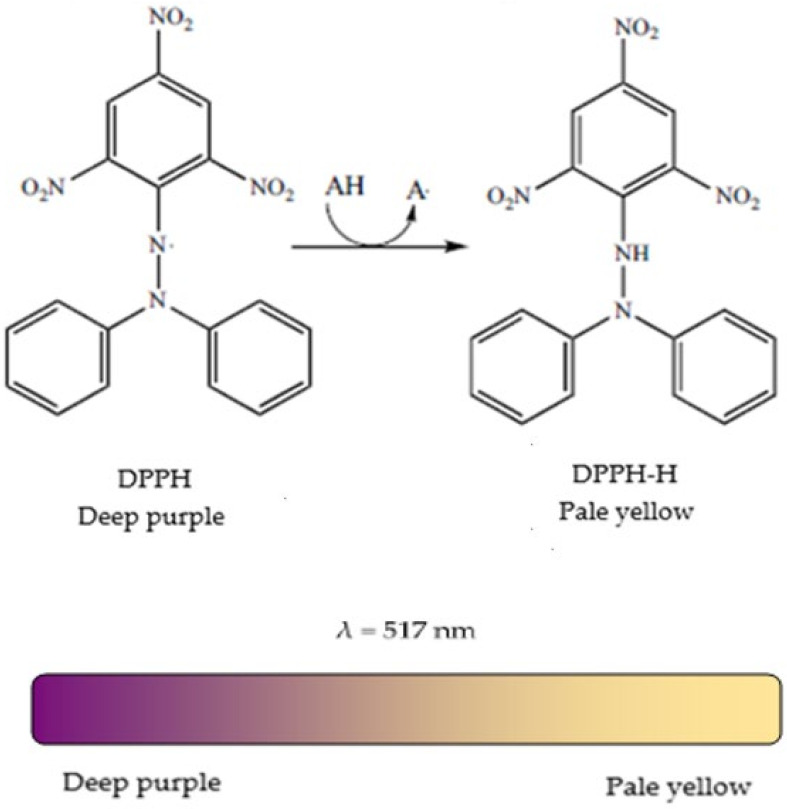
DPPH scavenging mechanisms by an antioxidant (AH).

The neutralisation DPPH test is based on donating electrons from the antioxidants in order to neutralise the DPPH radical. The reaction is accompanied by changing the DPPH colour measured at 517 nm, and discoloration acts as an indicator of antioxidant activity (Figure 10). Antioxidant activity by the DPPH neutralisation method is often reported as EC_50_, which is defined as the efficient concentration of the antioxidant necessary to reduce the initial DPPH concentration by 50%. In addition, T_EC50_ may be used, which is the necessary time to reach the equilibrium state with EC_50_ [92].

The DPPH test is a simple technique and requires only a Vis spectrophotometer or an electronic paramagnetic resonance (EPR) spectrometer. However, DPPH^•^ is not a natural radical but the mechanism of reaction with antioxidants is similar to that with peroxyl radicals ROO^•^ [93].

- Analytical protocols involving DPPH radical are varied. Drawing up an analytical protocol seems to be a simple procedure. The radical is freely soluble in methanol or ethanol, solvents that also dissolve most of the antioxidants which are of technological or functional interest. However, many other factors, such as the reaction period (fixed time or kinetic study), temperature (room or high temperature), and result expression are among the analytical variables that are extensively studied. Numerous reviews and research articles mention analytical details, protocol variations and innovations like changing the reaction monitoring from 515 to 580 nm in order to minimise interferences due to carotenoids [94], separation from radicals of the compounds tested in order to avoid spectral overlap by means of HPLC-PDA detectors [95] or HPLC postcolumn configuration that allows the estimation of the total antioxidant activity of the individual compounds in a mixture [96,97]. To evaluate the activity of radical elimination for compounds or extracts, the protocol proposed by Brand-Williams et al. (1995) remains the point of reference [9].
- Test users may be divided into two major groups: those who apply the test as a simple, quick, adequate test for the comparative evaluation of compounds or extracts, and those interested in the mechanistic aspects/the structure–activity relations of the antioxidants or a significant evaluation of the activity of an extract.
- An alternative proposal was made by Cheng et al. (2006) for the measurement of the area below the decomposition curve of DPPH^•^ (AUC) [98], an approach already used for other antioxidant activity tests, like the absorption capacity of the oxygen radicals (ORAC) [8]. In this manner, the kinetic and thermodynamic properties of the reaction were considered, and the results were expressed in relation to the AUC of a reference product.
- Test adjustments were proposed by various authors in an attempt to minimise issues related to test sensitivity and to simplify and automate the method [96]. Musa et al. (2013) investigated the use of the DPPH dry agent as an alternative to the freshly prepared DPPH in an automated test based on microplates. The modified method was validated by means of the classic DPPH test and, according to the authors, showed excellent sensitivity, being simpler to operate and more convenient with a minimum necessary amount of solvent [99].
- The neutralisation of the DPPH radical may be monitored by amperometric detection. When voltage is applied, the DPPH radical generates a constant electrical current, and the decrease in the radical amount as a result of neutralisation by the antioxidants results in a decrease in the amperometric signal. Thus, antioxidant quantification is performed by the amperometric detection of the residual concentration of unreacted DPPH radical. Sireerat and Schulte (2012) measured the antioxidant activity of tea infusions, fruit juices and vegetable extracts by means of an automated amperometric DPPH test, in which the measurements of antioxidant capacity were performed using the DPPH radical as a redox amperometric indicator. A Pb pencil was used as a working electrode, the counter-electrode was the platinum electrode, and the reference electrode was the Ag/AgCl electrode. This method was validated by synthetic antioxidants with known concentrations [100].
- The DPPH method was used to evaluate the antioxidant capacity of phenolic compounds [101], while Sun-Kun Yim et al. devised a continuous spectrophotometric test for reducing DPPH by NADPH (reduced form of Nicotinamide adenine dinucleotide phosphate)-cytochrome P450 reductase (CPR) in a mixed ethanol–water solution [102].

The reaction of uric acid with DPPH incorporated in micelles was explained by E. Abuin et al. [103], while D. Bandoniene and his colleagues [104] detected the compounds’ activity of radical elimination in a mixed ethanol–water solution by means of the DPPH and HPLC–DPPH method. To study the oxidation kinetics of aminoacids, G. Ionita et al. used a water-soluble DPPH derivative [105].

There were also reports of electrochemical methods for the measurement of antioxidant activity which are based on biosensors using cyclic voltammetry as a detection technique [106] to measure the antioxidant capacity of tert-butyl-hydroxyanisole [107]. These were used to study the efficiency of some antioxidants with a low molecular weight.

Another method of determining antioxidant activity based on the amperometric reduction of DPPH at the glassy carbon electrode was described by S. Milardović et al. [108]. The method was applied to the evaluation of the antioxidant activity of certain pure compounds, soluble in water or ethanol, certain antioxidants, and some samples of tea, wine and other beverages. The good correlation of measurements (R^2^ = 0.9993), expressed as trolox equivalent, was obtained by the amperometric method proposed and the classic spectroscopic method.

Of late, the DPPH test has been re-evaluated by Xie and Schaich (2014) for use in determining antioxidant activity by kinetic and stoichiometric approaches. The authors questioned the application of the DPPH test to classify antioxidants, and suggested it was possible to use it to distinguish the reaction mechanism of an antioxidant, i.e., SET and HAT, which display different reaction rates and models in various solvents [109].

Masek et al. (2016) researched the antioxidant activity of compounds of vegetable origin from the class of hydroxycynamic acids, by means of electrochemical methods, and the ABTS and DPPH tests [110]. The potential of these natural compounds to reduce iron and copper ions was measured by spectrophotometric analysis based on the FRAP and CUPRAC methods. Both methods, viz. electrochemical and spectrophotometric, allowed the qualitative analysis of phytochemical samples.

El Moussaoui et al. (2019) evaluated the antibacterial, antifungal and antioxidant activity in the root and leaves of the *Withania frutescence* species. The polyphenolic content was determined by the Folin–Ciocalteu reaction, and the measurement of the antioxidant activity was performed by four methods: the DPPH test, the FRAP test, total antioxidant capacity, and the β-carotene discolouration test [111].

The DPPH test is commonly applied to assess the antioxidant activity of plant extracts. To test the possible borate interference on the DPPH test, X. Chen et al. (2020) studied the effects of borates (0–16 mM sodium tetraborate) on the radical neutralisation capacity of Gallic acid (GA, at 10, 20 and 40 mg/L). The borate presence led to a significant decrease in DPPH inhibition by the GA, and its DPPH radical neutralisation capacity may be diminished according to the borate content. This interference was interpreted as resulting from GA auto-oxidation, as well as the formation of the ester complex GA–borate at alkaline pH (~9.50). Natural extracts of polyphenol from apple peels, parsley leaves, and green lettuce (as examples of potentially high borate content) also showed a low neutralisation capacity of the DPPH radical when enriched with borate. To avoid this drawback, a proposal was made to use the acetate tampon of pH 5.50 in preparing the samples. This paper highlighted the possible underestimation of the antioxidant properties of polyphenol extracts mainly derived from plants pre-treated with enriched boron or borate through the DPPH test [112].

##### Advantages of The DPPH Method

The application of this test allows the comprehension of the various chemical phenomena and has obvious advantages, like low cost, ease of performing experiments, reproducibility, applicability at room temperature, as well as automation possibilities. It explains why the scientific community still applies the DPPH^•^ test and is working on optimising/standardising protocols to produce significant and comparable results.

## 5. Conclusions and Future Trends

Antioxidants are becoming ever more interesting to scientists in the food field and medical professionals due to their protective roles in food products against oxidative deterioration and in the body against oxidative stress-mediated pathological processes. The efficient research of the sources of natural antioxidants and designing new antioxidant compounds require reliable methods of antioxidant activity evaluation.

Conventional methods for the measurement of antioxidant activity are still needed and specific methodological protocols are complex and require a long testing time. One of the important selection parameters of the antioxidant test is the working pH. There are tests operating in acidic (FRAP), neutral (CUPRAC) or alkaline (Folin−Ciocalteu) conditions.

Additionally, the applicability of the antioxidant test to both hydrophilic and lipophilic antioxidants is an important factor. While the ABTS and CUPRAC tests can measure both hydrophilic, and lipophilic antioxidants, some methods only measure hydrophilic antioxidants (FRAP and Folin−Ciocalteu), and others only apply to hydrophobic systems (DPPH).

At the same time, the background colour in the food matrix may trigger absorbance modifications, which have more significant adverse effects in the case of discolouration reactions (ABTS, DPPH), as compared to colour-formation reactions (FRAP, CUPRAC) [9].

Consequently, there is enormous potential in this research area, for the purpose of developing novel analytical methods of determining the compounds’ antioxidant capacity, especially in food products. For example, the development of electrochemical biosensors and the use of these in antioxidant research could be of great interest and could help in the study of the process kinetics. The great number of elements used in biological recognition, such as enzymes, aptamers, DNA/RNA and entire cells are the key elements in developing electrochemical biosensors applicable in the study of antioxidants. The advantages of biosensors in the study of antioxidants from complex samples are the portability, the fast measurement and the use of a small sample amount.

## Figures and Tables

**Table 1:** Reactive oxygen species (ROS) and non free-radical species.

Reactive Oxygen Species		Non Free-Radical Species	
Hydroxyl radical	HO^•^	Hydrogen peroxide	H_2_O_2_
Superoxide radical	O_2_^•^	Singlet oxygen	^1^O_2_
Hydroperoxyl radical	HOO^•^	Ozone	O_3_
Lipid radical	L^•^	Lipid hydroperoxide	LOOH
Lipid peroxyl radical	LOO^•^	Hypochlorous acid	HOCl
Peroxyl radical	ROO^•^	Peroxynitrite	ONOO^−^
Lipid alkoxyl radical	LO^•^	Dinitrogen trioxide	N_2_O_3_
Nitrogen dioxide radical	NO_2_^•^	Nitrous acid	HNO_2_
Nitric oxide radical	NO^•^	Nitryl chloride	NO_2_Cl
Thiyl radical	RS^•^	Nitroxyl anion	NO^−^
Protein radical	P^•^	Nitrosyl cation	NO^+^

**Table 2:** Different techniques used to measure antioxidant activity.

Techniques	Antioxidant Capacity Assay		Principle of the Method	End-Product Determination
**Spectrometry**		ORAC	Antioxidant reaction with peroxyl radicals, induced by 2,2′-azobis-2-amidino-propane (AAPH)	Loss of fluorescence of fluorescein
		HORAC	Antioxidant capacity to quench OH radicals generated by a Co(II) based Fenton-like system	Loss of fluorescence of fluorescein
		TRAP	Antioxidant capacity to scavenge luminol-derived radicals, generated from AAPH decomposition	Chemiluminescence quenching
		CUPRAC	Cu (II) reduction to Cu (I) by antioxidants	Colorimetry
		FRAP	Antioxidant reaction with a Fe(III) complex	Colorimetry
		PFRAP	Potassium ferricyanide reduction by antioxidants and subsequent reaction of potassium ferrocyanide with Fe^3+^	Colorimetry
		ABTS	Antioxidant reaction with an organic cation radical	Colorimetry
		DPPH	Antioxidant reaction with an organic radical	Colorimetry
	**Fluorimetric** **Analysis**		Emission of light by a compound, which has absorbed light or other electromagnetic radiation of a different wavelength	Recording of fluorescence excitation/emission spectra
Electrochemical Techniques		Voltammetry	The reduction or oxidation of a compound at the surface of a working electrode, at the appropriate applied potential, resulting in the mass transport of new material to the electrode surface and in the generation of a current	Measurement of the current of the cathodic/anodic peak
		Amperometry	The potential of the working electrode is set at a fixed value with respect to a reference electrode	Measurement of the current generated by the oxidation/ reduction of an electroactive analyte
		Biamperometry	The reaction of the analyte (antioxidant) with the oxidised form of a reversible indicating redox couple	Measurement of the current flowing between two identical working electrodes, at a small potential difference and immersed in a solution containing the analysed sample and a reversible redox couple
Chromatography		Gas chromatography	Separation of the compounds in a mixture is based on the repartition between a liquid stationary phase and a gas mobile phase	Flame ionisation or thermal conductivity detection
		High performance liquid chromatography	Separation of the compounds in a mixture is based on the repartition between a solid stationary phase and a liquid mobile phase with different polarities, at high flow rate and pressure of the mobile phase	UV-Vis (e.g., diode array) detection, fluorimetric detection, mass spectrometry or electrochemical detection

ORAC—Oxygen Radical Absorption Capacity; HORAC—Hydroxyl Radical Antioxidant Capacity; TRAP—Total Peroxyl Radical Trapping Antioxidant Parameter; CUPRAC—Cupric Reducing Antioxidant Power; FRAP—Ferric Reducing Antioxidant Power; PFRAP—potassium ferricyanide reducing power; ABTS—2,2′-Azinobis-(3-ethylbenzothiazoline-6-sulfonic acid; DPPH—[2,2-di(4-tert-octylphenyl)-1-picrylhydrazyl].
